# Women’s reproductive span: a systematic scoping review

**DOI:** 10.1093/hropen/hoac005

**Published:** 2022-02-11

**Authors:** A F Nabhan, G Mburu, F Elshafeey, R Magdi, M Kamel, M Elshebiny, Y G Abuelnaga, M Ghonim, M H Abdelhamid, Mo Ghonim, P Eid, A Morsy, M Nasser, N Abdelwahab, F Elhayatmy, A A Hussein, N Elgabaly, E Sawires, Y Tarkhan, Y Doas, N Farrag, A Amir, M F Gobran, M Maged, M Abdulhady, Y Sherif, M Dyab, J Kiarie

**Affiliations:** Department of Obstetrics and Gynecology, Faculty of Medicine, Ain Shams University, Cairo, Egypt; The UNDP/UNFPA/UNICEF/WHO/World Bank Special Programme of Research, Development and Research Training in Human Reproduction (HRP Research), World Health Organization, Geneva, Switzerland; Egyptian Center for Evidence Based Medicine, Cairo, Egypt; Egyptian Center for Evidence Based Medicine, Cairo, Egypt; Egyptian Center for Evidence Based Medicine, Cairo, Egypt; Egyptian Center for Evidence Based Medicine, Cairo, Egypt; Egyptian Center for Evidence Based Medicine, Cairo, Egypt; Egyptian Center for Evidence Based Medicine, Cairo, Egypt; Egyptian Center for Evidence Based Medicine, Cairo, Egypt; Egyptian Center for Evidence Based Medicine, Cairo, Egypt; Egyptian Center for Evidence Based Medicine, Cairo, Egypt; Egyptian Center for Evidence Based Medicine, Cairo, Egypt; Egyptian Center for Evidence Based Medicine, Cairo, Egypt; Egyptian Center for Evidence Based Medicine, Cairo, Egypt; Egyptian Center for Evidence Based Medicine, Cairo, Egypt; Egyptian Center for Evidence Based Medicine, Cairo, Egypt; Egyptian Center for Evidence Based Medicine, Cairo, Egypt; Egyptian Center for Evidence Based Medicine, Cairo, Egypt; Egyptian Center for Evidence Based Medicine, Cairo, Egypt; Egyptian Center for Evidence Based Medicine, Cairo, Egypt; Egyptian Center for Evidence Based Medicine, Cairo, Egypt; Egyptian Center for Evidence Based Medicine, Cairo, Egypt; Egyptian Center for Evidence Based Medicine, Cairo, Egypt; Egyptian Center for Evidence Based Medicine, Cairo, Egypt; Egyptian Center for Evidence Based Medicine, Cairo, Egypt; Egyptian Center for Evidence Based Medicine, Cairo, Egypt; Egyptian Center for Evidence Based Medicine, Cairo, Egypt; The UNDP/UNFPA/UNICEF/WHO/World Bank Special Programme of Research, Development and Research Training in Human Reproduction (HRP Research), World Health Organization, Geneva, Switzerland

**Keywords:** reproductive span, menarche, menopause, demography, assisted reproduction, infertility, humans, female

## Abstract

**STUDY QUESTION:**

What is the scope of literature regarding women’s reproductive span in terms of definitions, trends and determinants?

**SUMMARY ANSWER:**

The scoping review found a wide variation in definitions, trends and determinants of biological, social and effective women’s reproductive span.

**WHAT IS KNOWN ALREADY:**

A woman’s reproductive span refers to her childbearing years. Its span influences a woman’s reproductive decisions.

**STUDY DESIGN, SIZE, DURATION:**

A systematic scoping review was conducted. We searched MEDLINE, PubMed, JSTOR, CINAHL, Web of Science and Scopus electronic databases from inception to January 2021 without imposing language or date restrictions. We searched unpublished sources including the Global Burden of Disease, Demographic and Health Surveys, and National Health and Nutrition Examination Surveys. The list of relevant references was searched by hand. Sixty-seven reports on women’s reproductive span were included in this review.

**PARTICIPANTS/MATERIALS, SETTING, METHODS:**

This scoping systematic review followed an established framework. The reporting of this scoping review followed the reporting requirements provided in the Preferred Reporting Items for Systematic Reviews and Meta-Analyses, Extension for Scoping Reviews. Identified records were independently screened and data were extracted. We performed conceptual synthesis by grouping the studies by available concepts of reproductive span and then summarized definitions, measures used, temporal trends, determinants, and broad findings of implications on population demographics and assisted reproduction. Structured tabulation and graphical synthesis were used to show patterns in the data and convey detailed information efficiently, along with a narrative commentary.

**MAIN RESULTS AND THE ROLE OF CHANCE:**

A total of 67 relevant reports on women’s reproductive span were published between 1980 and 2020 from 74 countries. Most reports (42/67) were cross-sectional in design. Literature on reproductive span was conceptually grouped as biological (the interval between age at menarche and age at menopause), effective (when a woman is both fertile and engaging in sexual activity) and social (period of exposure to sexual activity). We summarized the working definitions, trends and determinants of each concept. Few articles addressed implications on demographics and assisted reproduction.

**LIMITATIONS, REASONS FOR CAUTION:**

A formal assessment of methodological quality of the included studies was not performed because the aim of this review was to provide an overview of the existing evidence base regardless of quality.

**WIDER IMPLICATIONS OF THE FINDINGS:**

The review produced a comprehensive set of possible definitions of women’s reproductive span, trends, and potential determinants. Further advancement of these findings will involve collaboration with relevant stakeholders to rate the importance of each definition in relation to demography and fertility care, outline a set of core definitions, identify implications for policy, practice or research and define future research opportunities to explore linkages between reproductive spans, their determinants, and the need for assisted reproduction.

**STUDY FUNDING/COMPETING INTEREST(S):**

This work received funding from the UNDP-UNFPA-UNICEF-WHO-World Bank Special Programme of Research, Development and Research Training in Human Reproduction (HRP), a cosponsored programme executed by the World Health Organization (WHO). The authors had no competing interests.

**STUDY REGISTRATION NUMBER:**

N/A.

WHAT DOES THIS MEAN FOR PATIENTS?

A woman’s ‘reproductive span’ is an important concept that includes her childbearing years and therefore has an impact on her decision making, including when to try for a pregnancy, spacing between pregnancies, desired family size and, finally, when to have the last baby. There have been notable changes in recent decades, with women choosing to delay marriage, not to marry at all, postpone childbearing or limit the number of births. This study searched for all published research on women’s reproductive span. Studies were grouped as biological (the interval between the beginning and end of menstruation), effective (when a woman is both fertile and sexually active) and social (period of exposure to sexual activity). Currently, the biological reproductive span of women ranges from 30.9 to 39.3 years, while the effective reproductive span was found to vary, with a steady decline worldwide. A wide variety of determinants of the reproductive span were reported in the literature, but limited studies reported the implications of contemporary trends in reproductive span on population demographics or assisted reproduction. Trends in women’s reproductive span may have an impact on the need or utilization of fertility care services, including medically assisted reproduction.

## Introduction

Globally, infertility is considered a major public health issue, affecting ∼8–12% of couples or 186 million people (Inhorn and Patrizio, 2015; Vander Borght and Wyns, 2018). Infertility remains a woman’s social burden (Inhorn and Patrizio, 2015), affecting 8% of women aged 19–26 years, 13–14% of women aged 27–34 years and 18% of women aged 35–39 years (Dunson *et al.*, 2004). Although advances in reproductive medicine continuously provide additional solutions and interventions for those who desire to conceive, an important challenge that remains is that women have a finite reproductive lifespan (Inhorn and Patrizio, 2015).

A woman’s reproductive span is an important concept that encompasses childbearing years and therefore has an impact on women’s reproductive decisions including when to get pregnant, spacing between pregnancies, desired family size and, finally, when to have the last birth. With notable changes in social-economic contexts over the past decades, more women may choose to delay marriage, not to marry at all, postpone childbearing to an older age or limit the number of births. Since women’s fertility declines with age owing to a decline in the number and quality of oocytes, the propensity to delay childbearing has a significant impact on fertility because it reduces the number of reproductive years, particularly the most fertile years (Velde and Pearson, 2002).

Under most demographic circumstances, reproduction during this period in a woman’s life is the most important determinant of population dynamics and growth (Vitzthum, 2021). Therefore, advancing our understanding of women’s reproductive span and its determinants and trends is critical for making future directions for policy, practice and research (Carey and Roach, 2020).

The rationale to conduct this scoping review was based on the absence of any publication examining the scope of literature on women’s reproductive span.

The aim of this systematic scoping review was, therefore, to determine the scope of literature and to synthesize what is known about women’s reproductive span in terms of definitions, trends and determinants, and the impact that contemporary trends in reproductive span have on population demographics and assisted reproduction.

## Materials and methods

A scoping review approach was chosen as the appropriate method, given the broad and complex nature of the concept of women’s reproductive span. To confirm that no other similar scoping reviews existed, Medline and Prospero databases were searched, and the results indicated an absence of systematic scoping articles related to women’s reproductive span. The review was conducted based on the methods that were pre-specified in the protocol. The review protocol was prospectively registered in the Open Science Framework platform (https://osf.io/wysru; Nabhan *et al.*, 2020).

The methods for this scoping review were guided by the framework developed by Arksey and O’Malley (2005), subsequently adapted by Levac *et al.* (2010), Colquhoun *et al.* (2014) and by the Joanna Briggs Institute guidelines (Peters *et al.*, 2015), as described below, in five stages.

- Stage 1: Identifying research questions. The following questions guided the scoping review: What are the definitions of the reproductive span? What are the trends in the reproductive span? What are the determinants of the reproductive span? What are the effects of the reproductive span on population demography? What are the effects of the reproductive span on fertility services?
- Stage 2: Identifying relevant studies. We conducted a systematic search to identify both published and unpublished sources relevant to the concept of women’s reproductive span.

As a first step, an initial limited search of one bibliographic database was performed. We analyzed the text words contained in the titles, abstracts and index terms in the retrieved articles. In the second step, all identified text words and index terms were used to develop the search strategy by an experienced author [A.F.N.]. The search strategy was further refined through team discussion. The strategy for searching bibliographic databases included the following terms ‘menopause/statistics and numerical data’ [MeSH Terms] OR ‘menarche/statistics and numerical data’ [MeSH Terms] OR ‘age at menarche’ [Text Word] OR ‘age at menopause’ [Text Word] OR ‘age at natural menopause’ [Text Word] OR ‘reproductive span’ [Text Word]. The search strategy for different databases can be found in Supplementary Data. We searched MEDLINE, PubMed, JSTOR, CINAHL, Web of Science and Scopus electronic databases from inception to January 2021. The search was updated in December 2021. We also searched the Fertility Estimates 1950–2019 and Population Estimates 1950–2019 of the Global Burden of Disease Study 2019, Organization for Economic Co-operation and Development Database, Demographic and Health Surveys data sets and the National Health and Nutrition Examination Survey data sets. We did not impose any language or date restriction. In the third step, for all relevant articles, we hand-searched the list of references and explored the cited-by logs.

**Figure 1.**
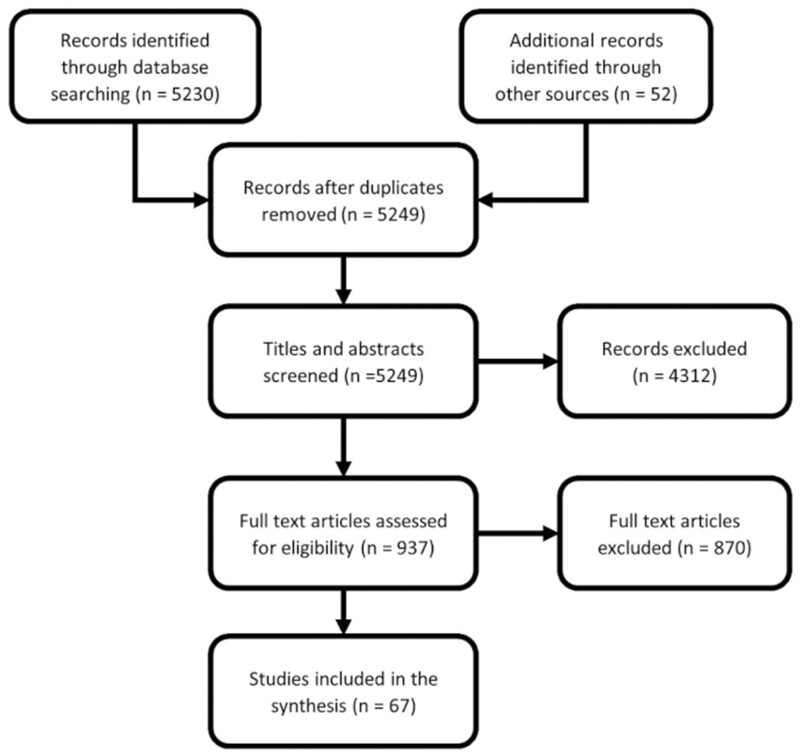
**PRISMA flowchart.** PRISMA, Preferred Reporting Items for Systematic Reviews and Meta-Analyses, Extension for Scoping Reviews.

- Stage 3: Study selection. Inclusion criteria were studies that reported on women’s (population) reproductive span (concept) and from any country globally (context). All study designs were eligible. The titles and abstracts of the records identified by electronic search were independently screened by two authors. This was followed by reviewing the full text of potentially relevant articles. If an agreement for inclusion could not be reached between the two authors, an opinion was requested from a third author. Figure 1 shows the process of study selection.
- Stage 4: Data charting process. A data extraction form was developed *a priori* to capture relevant data from included studies. It was piloted and refined based on feedback from the team during regular meetings. The team regularly discussed the data and continuously updated the data-charting form in an iterative process. Two authors independently extracted the following data items: report data (title (TI), publication date (DP), first author (FAU), language (LA), publication type (PT), article identifier (AID)), methodological data (research design, participants, sample if applicable, study period, countries), definitions of reproductive span, data used for estimating the reproductive span, temporal trends and implications on population demographics and assisted reproduction. We did not plan to perform a formal critical appraisal of studies for this scoping review.
- Stage 5: Collating and summarizing results. We performed conceptual synthesis by grouping the studies by concepts and then summarized definitions, measures used, temporal trends, determinants and broad findings of implications on population demographics and assisted reproduction. Structured tabulation and graphical synthesis were used to show patterns in the data and convey detailed information efficiently along with a narrative commentary.

The review was reported in accordance with the reporting guidance provided in the Preferred Reporting Items for Systematic Reviews and Meta-Analyses, Extension for Scoping Reviews (PRISMA-ScR) (Tricco *et al.*, 2018).

R software v4 was used for text mining, data wrangling and data visualization (R Core Team, 2020).

## Results

### Literature search results

The electronic search yielded 5230 records and an additional 52 records from hand searches. Screening titles and abstracts identified 937 potentially relevant records. These potentially relevant full-length articles were assessed, and 67 sources were included in this scoping review as depicted in the PRISMA flowchart (Fig. 1). We further explored two data sets (‘UK Biobank,’ 2021; ‘Centers for Disease Control and Prevention (CDC).’ n.d.) and one dissertation (Mulder, 1987) for additional data related to the included publications. Reports were excluded if they did not contain data on women’s reproductive span.

### Mapping of research findings

#### Study design

The literature included studies with different methodologies. The majority (42/67; 62.69%) used a cross-sectional study design (Table I). The publication date of the included studies extended from 1980 to 2020.

**Table I:** Different methodologies used in the literature on women’s reproductive span.

Design	Count
Case–control	4
Cohort	
Ambidirectional	1
Prospective	3
Retrospective	6
Cross-sectional	42
Reviews	
Meta-analysis	3
Narrative Review	5
Systematic Review	1
Systematic review and meta-analysis	2

#### Participants

The extent of the literature on women’s reproductive span encompassed participants across all races, ethnic groups, ancestries, religions, socioeconomic status, residence, marital status, educational levels and occupations. The age of participants ranged from 3 to 89 years, with birth cohorts and women born as early as 1900.

#### Context

**Figure 2.**
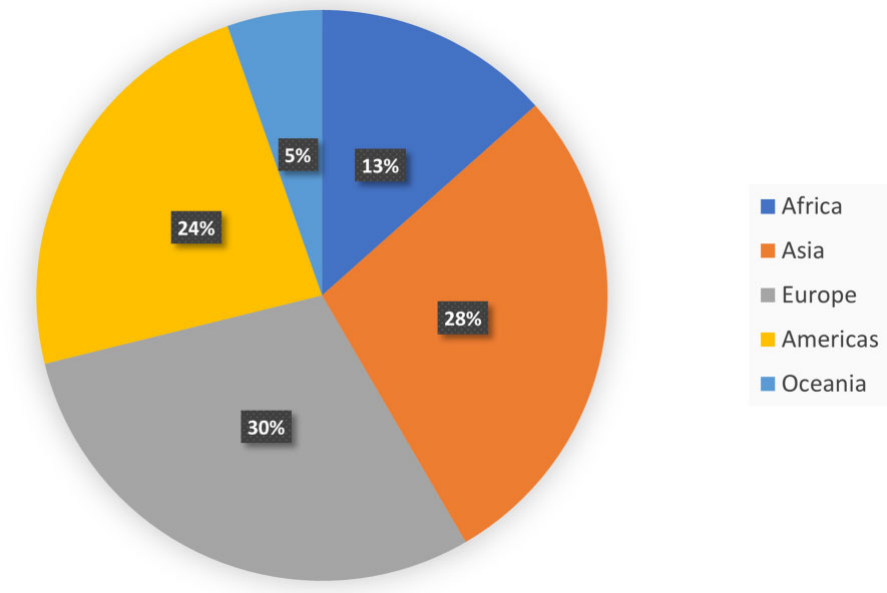
Available literature identified by this scoping review on women’s reproductive span, shown as percentage of available datasets from each continent.

All continents contributed data to the literature on women’s reproductive span with 44 data sets from Europe, 42 from Asia, 35 from Americas, 20 from Africa and 8 from Oceania (Table II), (Figure 2). Data were available from 74 countries. USA, India and China contributed the largest number of studies on women’s reproductive span.

**Table II:** Regions and sub-regions contributing to the literature on women’s reproductive span.

Region	Sub-region	Data sets
Africa	Northern Africa	5
	Sub-Saharan Africa	15
Americas	Latin America and the Caribbean	16
	Northern America	19
Asia	Eastern Asia	16
	South-eastern Asia	7
	Southern Asia	13
	Western Asia	6
Europe	Eastern Europe	6
	Northern Europe	18
	Southern Europe	8
	Western Europe	12
Oceania	Australia and New Zealand	5
	Melanesia	2
	Polynesia	1

#### Concept

Conceptual synthesis of reproductive span included biological, effective and social (Table III).

**Table III:** Mapping different concepts and working definitions used in the literature on women’s reproductive span.

Concept	Starts at	Ends at	Literature
Biological	Age at menarche	Age at menopause	Singh and Ahuja (1980), Beall (1983), Goodman et al. (1985), Kapoor and Kapoor (1986), Menken (1987), Wood and Weinstein (1988), Thomas et al. (2001), Padmadas et al. (2004), Riener et al. (2004), Aydos et al. (2005), Kalichman et al. (2007); Dorjgochoo et al. (2008), Liu et al. (2010), Lu et al. (2010), Cerne et al. (2011) Fukuda et al.,(2011), Forman et al. (2013), Tea et al. (2013), Pyun et al. (2014), Duarte et al. (2017), Bjelland et al. (2018), Shaw et al. (2018), Demakakos et al. (2019), Gottschalk et al. (2020) Singh et al. (2020) and Sinha et al. (2021)
	Age at menarche	Age at natural menopause	Morabia et al. (1996), Morabia and Costanza (1998), Johnston (2001), Hefler et al. (2002), Worda et al. (2004), Long et al. (2006), Nichols et al. (2006), Zerbetto et al. (2008), Hartge (2009), He et al. (2009a, 2010), Chen et al. (2010), Barlow (2011), Yunus et al. (2014), Shi et al. (2016), Bjelland et al. (2018), Fernández-Rhodes et al. (2018) and Gottschalk et al. (2020)
	Age at menarche	Age at induced menopause	Snieder et al. (1998), Nichols et al. (2006), Barlow (2011), Chen et al. (2012), Carty et al. (2013), and Bjelland et al. (2018)
Effective	Age at first marriage	Age at menopause or age at sterilization of the woman or her spouse	Padmadas et al. (2004) and Singh et al. (2020)
	Age at marriage	Age at menopause or age at sterilization of the woman	Singh and Singh (2014)
	Age at marriage	Age at sterilization of the woman or her spouse	Murthy (2012a,b)
	Age at first marriage or menarche, whichever occurs last	Age at menopause or marriage dissolution, whichever occurs first	Menken (1987)
	Age at marriage	Age at sterilization of the woman	Wood et al. (1985)
	Age at marriage	Age at last live birth	Mulder (1989) and Singh et al. (2020)
	Estimated age at menarche or age at cliteroidectomy minus 6 months	Age at last live birth	Mulder (1989)
	Age at first birth	Age at last live birth	Horne (1989), Stevenson et al. (1989) and Singh et al. (2020)
Social	Age at marriage or entry into a union in which sexual relations take place regularly	Age at marriage dissolution or permanent abstinence	Menken (1987), Padmadas et al. (2004) and Singh et al. (2020)
	Age at which both partners cohabit (approximately a year after marriage)	Age at marriage dissolution (widowhood)	Wood et al. (1985)

### Definitions and measures of reproductive span

#### Biological reproductive span

Studies used different terms for the ‘biological reproductive span’ (Beall, 1983; Menken, 1987; Padmadas *et al.*, 2004; Barlow, 2011), including ‘reproductive period’ (Riener *et al.*, 2004; Liu *et al.*, 2010; Cerne *et al.*, 2011; Yunus *et al.*, 2014; Bjelland *et al.*, 2018), ‘menstruation span’ (Chen *et al.*, 2010), ‘reproductive years’ (Nichols *et al.*, 2006; Dorjgochoo *et al.*, 2008; Forman *et al.*, 2013), ‘fertile span’ (Goodman *et al.*, 1985), ‘total fertility span’ (Kapoor and Kapoor, 1986), ‘years of menstruation’ (Long *et al.*, 2006), ‘reproductive life’ (Morabia *et al.*, 1996; Morabia and Costanza, 1998), ‘potential span’ (Singh and Ahuja, 1980; Padmadas *et al.*, 2004; Singh *et al.*, 2020), ‘span of fertility’ (Shi *et al.*, 2016), ‘natural reproductive period’ (Thomas *et al.*, 2001; Sinha *et al.*, 2021), ‘fertile period’ (Tea *et al.*, 2013), ‘total years of fertility’ (Zerbetto *et al.*, 2008) and ‘menstrual life’ (Singh and Ahuja, 1980).

The biological reproductive span broadly constitutes the interval between age at menarche and age at menopause (Singh and Ahuja, 1980; Beall, 1983; Goodman *et al.*, 1985; Kapoor and Kapoor, 1986; Menken, 1987; Wood and Weinstein, 1988; Thomas *et al.*, 2001; Padmadas *et al.*, 2004; Riener *et al.*, 2004; Aydos *et al.*, 2005; Kalichman *et al.*, 2007; Dorjgochoo *et al.*, 2008; Liu *et al.*, 2010; Lu *et al.*, 2010; Cerne *et al.*, 2011; Fukuda *et al.*, 2011; Forman *et al.*, 2013; Tea *et al.*, 2013; Pyun *et al.*, 2014; Duarte *et al.*, 2017; Bjelland *et al.*, 2018; Shaw *et al.*, 2018; Demakakos *et al.*, 2019; Gottschalk *et al.*, 2020; Singh *et al.*, 2020; Sinha *et al.*, 2021). The end of the biological span might be age at natural menopause (Pavia *et al.*, 1994; Morabia *et al.*, 1996; Morabia and Costanza, 1998; Johnston, 2001; Hefler *et al.*, 2002; Worda *et al.*, 2004; Bartmann *et al.*, 2005; Long *et al.*, 2006; Nichols *et al.*, 2006; He *et al.*, 2007, 2009b; Kalichman *et al.*, 2007; Kevenaar *et al.*, 2007; Dorjgochoo *et al.*, 2008; Mitchell *et al.*, 2008; Zerbetto *et al.*, 2008; Hartge, 2009; He *et al.*, 2009a, 2010; Chen *et al.*, 2010; Liu *et al.*, 2010; Barlow, 2011; Cerne *et al.*, 2011; Fukuda *et al.*, 2011; Chen *et al.*, 2012; Carty *et al.*, 2013; Lewington *et al.*, 2014; Pyun *et al.*, 2014; Yunus *et al.*, 2014; Duan *et al.*, 2015; Ruth *et al.*, 2016; Shi *et al.*, 2016; Mishra *et al.*, 2017; Bjelland *et al.*, 2018; Fernández-Rhodes *et al.*, 2018; Huang *et al.*, 2018; Sharma and Bansal, 2018; Demakakos *et al.*, 2019; InterLACE Study Team, 2019; Gottschalk *et al.*, 2020; Sinha *et al.*, 2021) or surgically-, hormonally-, chemotherapy- or radiation-induced menopause (Chow *et al.*, 1997; Snieder *et al.*, 1998; Nichols *et al.*, 2006; Barlow, 2011; Chen *et al.*, 2012; Carty *et al.*, 2013; Bjelland *et al.*, 2018).

#### Social reproductive span

The social reproductive span is the period of exposure to sexual activity, defined as the duration between marriage or entry into a union in which sexual relations take place regularly and final marriage dissolution or permanent abstinence (Menken, 1987; Wood and Weinstein, 1988; Padmadas *et al.*, 2004; Singh *et al.*, 2020). While marriage dissolution entails separation of a couple or widowhood, permanent abstinence may be culturally dictated (Menken, 1987). In some cultures, the social reproductive span starts when both partners co-habit (approximately a year after marriage) and ends at widowhood, as there is no divorce once the first child is born (Wood *et al.*, 1985).

#### Effective reproductive span

The effective or behavioral (Singh *et al.*, 2020) reproductive span, during which a woman is both fertile and engaging in sexual activity, represents the overlap of the biological and social reproductive spans (Menken, 1987).

Effective span extends from the age at marriage or entry into a union in which sexual relations take place regularly to the age at menopause (Padmadas *et al.*, 2004; Singh and Singh, 2014; Singh *et al.*, 2020), from marriage until sterilization (Wood *et al.*, 1985; Padmadas *et al.*, 2004; Murthy, 2012a,b; Singh and Singh, 2014; Singh *et al.*, 2020), whether sterilization of either partner (Padmadas *et al.*, 2004; Murthy, 2012a,b; Singh *et al.*, 2020) or sterilization of the woman (Singh and Singh, 2014).

Other definitions included the years from the first marriage or menarche, whichever occurs last, to menopause or marriage dissolution, whichever occurs first (Menken, 1987), from marriage to last birth (Singh *et al.*, 2020) or from first birth to last birth (Horne, 1989; Stevenson *et al.*, 1989; Singh *et al.*, 2020). One study derived the effective reproductive span by two methods both having age at last livebirth as the endpoint, while the start point was either the age at marriage or an estimated age at menarche (Mulder, 1989).

### Temporal trends in women’s reproductive span

Data from recent datasets indicate that the duration of the biological reproductive span, worldwide, ranges from 30.94 to 39.30 years, with a mean (SD) of 35.85 (2.02) years.

**Figure 3.**
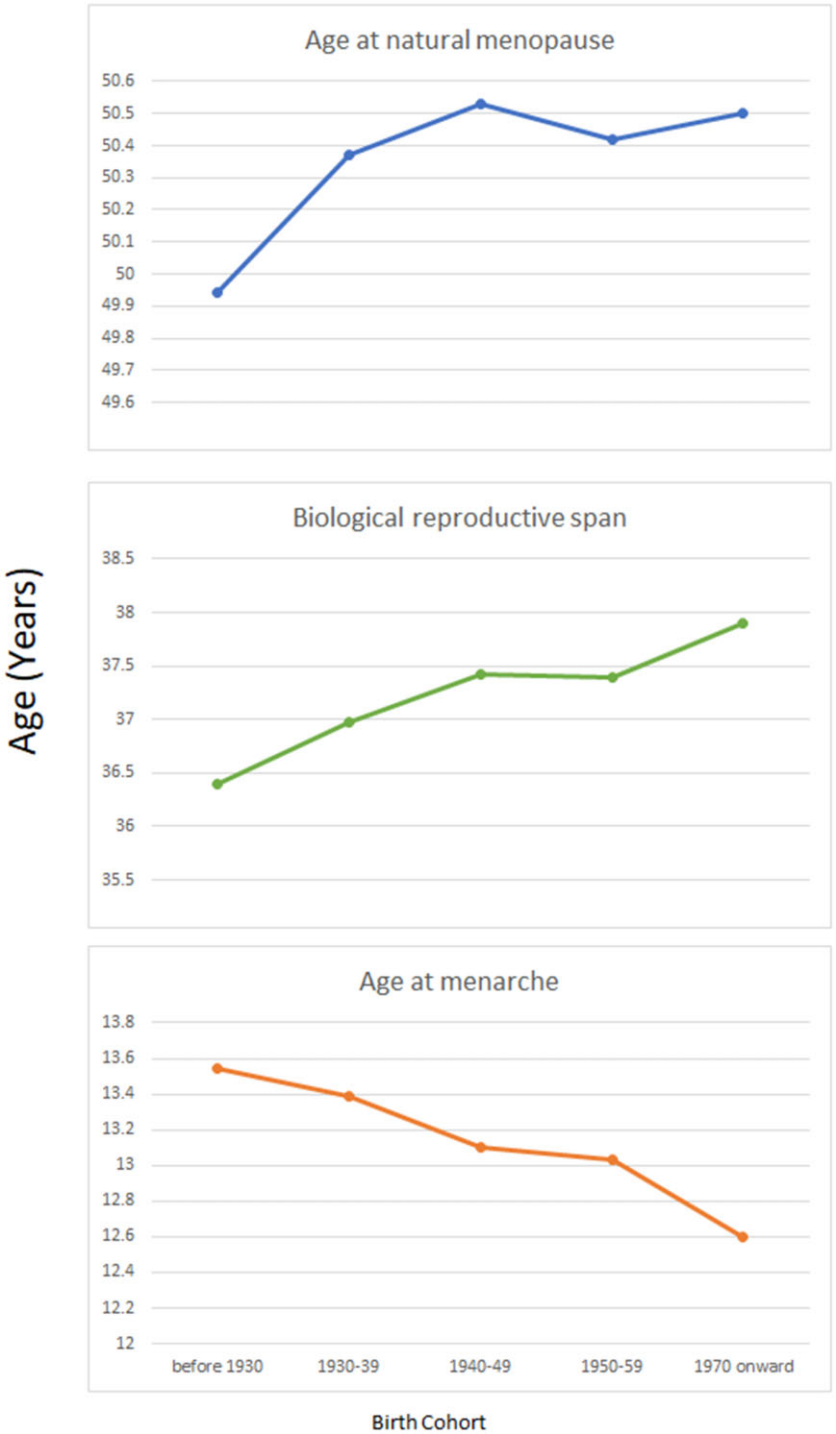
**Temporal trend of women’s biological reproductive span: pooled data from 23 studies across 10 countries.** Data points are mean values. The 10 countries are Australia, Demark, Sweden, Norway, UK, USA, Japan, Lebanon, Spain and Morocco.

Data from 23 studies across 10 countries (Australia, Demark, Sweden, Norway, UK, USA, Japan, Lebanon, Spain and Morocco) contributed to the estimates of age at menarche, age at first birth and age at menopause in women born between 1900 and 1984 (InterLACE Study Team, 2019) (Table IV). The mean age at menarche declined steadily from women born before 1930 to those born after 1970 (13.5 versus 12.6 years), the age at menopause remained steady with no significant change, the age at first birth, however, showed an initial decline from 1900 to 1949 (27.2 versus 24.8 years) followed by a progressive rise to 27.3 years for women born after 1970. The mean values for biological span increased from 36.4 to 37.9 years in women born before 1930 and those born after 1970, respectively. The mean values for effective span followed a trend, with an initial increase for women born between 1900 and 1949 (22.69 versus 25.25) followed by a decline for women born in 1970 onward (mean 23.12 years) (InterLACE Study Team, 2019) (Fig. 3).

**Table IV:** Temporal trend of women’s reproductive span: pooled data from 10 countries.

Birth cohort	Age at menarche	Age at natural menopause	Biological span	Age at first birth	Effective Span
Before 1930	13.54	49.94	36.40	27.25	22.69
1930–1939	13.39	50.37	36.98	26.26	24.11
1940–1949	13.10	50.53	37.43	25.25	25.28
1950–1959	13.03	50.42	37.39	25.81	24.61
1970 onward	12.60	50.50	37.90	27.38	23.12

All data are in years.

**Figure 4.**
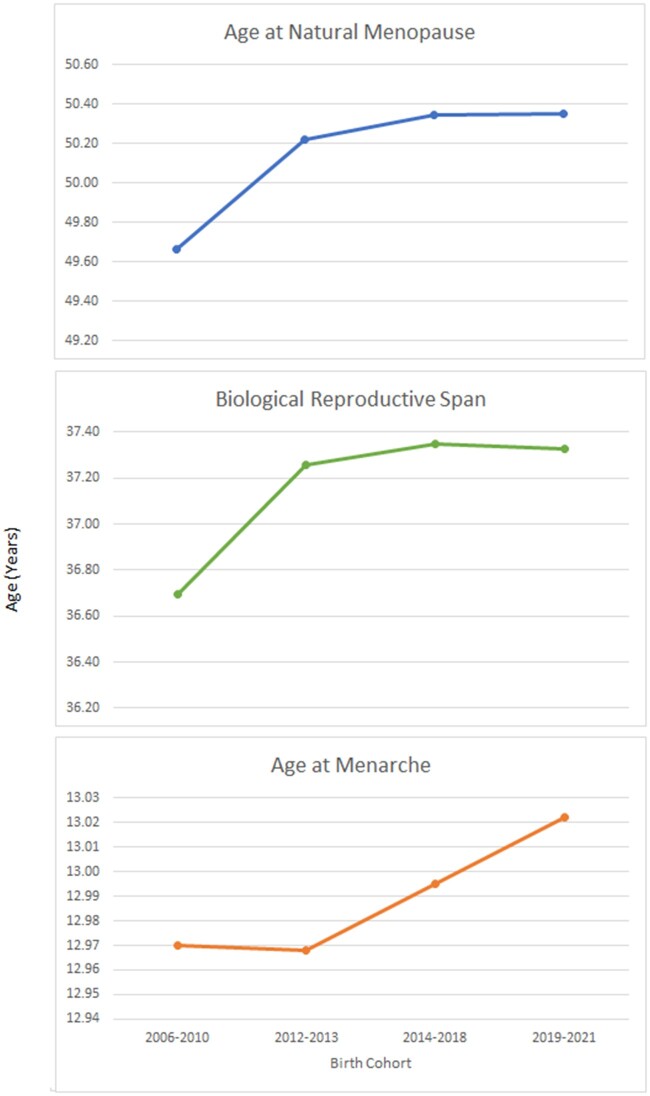
**Temporal trend of women’s biological reproductive span: UK data.** Data points are mean values.

**Figure 5.**
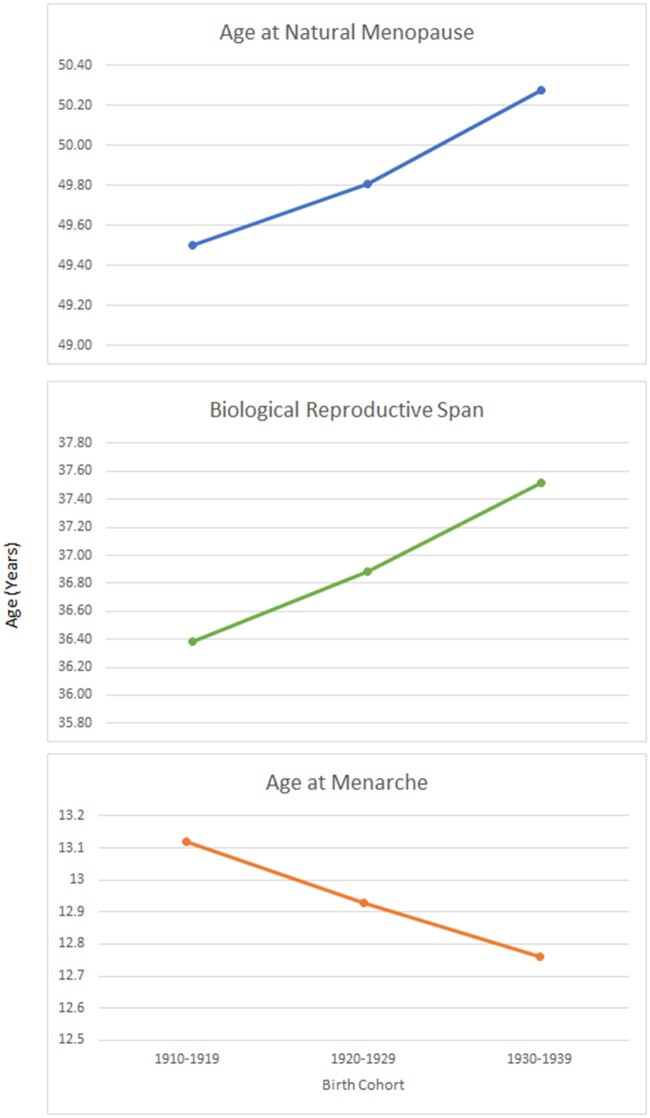
**Temporal trend of women’s biological reproductive span: US data.** Data points are mean values.

- China: data included 45 birth cohorts (born before 1930 to after 1970) in socially diverse urban and rural regions of China. The mean increased from 47.9 to 49.3 years. Mean age at menarche decreased steadily from 16.1 to 14.3 years. The biological reproductive span showed an increasing trend from 31.8 to 35 years (Lewington *et al.*, 2014).
- Norway: data included women born in Norway during the years 1936–1964. The mean age at menarche decreased from 13.42 years among women born during 1936–1939 to 13.24 years among women born during 1960–1964. The mean age at menopause increased from 50.31 years among women born during 1936–1939 to 52.73 years among women born during 1960–1964. The mean biological reproductive span increased from 36.83 years to 40.22 years (Gottschalk *et al.*, 2020).
- Russia: in a rural population, the mean values of age at menopause increased from 47.0 years (women born 1920–1925) to 49.7 years (women born 1940–1945) and 49.3 years (women born 1945–1950). Mean values of the biological reproductive span increased from 30.7 (women born 1920–1925) to 34.1 (women born during 1940–1945) and then slightly decreased to 33.7 years (women born 1945–1950) (Kalichman *et al.*, 2007).
- UK: for this review, we extracted available data from the UK Biobank (‘UK Biobank,’ 2021) from 2006 to 2019 (Table V). The biological and effective reproductive span remained stable from 2006 to 2019 onward (Fig. 4).
- USA: data collected between 1988 and 2001 included women born between 1910 and 1969. Birth cohorts were created using 5- and 10-year periods. The mean age at menarche decreased for those born between 1910 and 1939 (13.12 versus 12.76 years), with a subsequent increase to 13.0 years among women born between 1960 and 1969. Among naturally menopausal women aged 60 or more years, there was an increase in the mean age at menopause for those born between 1910 and 1939 (49.51 versus 50.28 years). Mean values of the biological reproductive span (subtracting age at menarche from age at menopause), increased from 36.4 years among women born between 1910 and 1919 to 37.5 years among the 1930–1939 cohort (Nichols *et al.*, 2006) (Table VI, Fig. 5).
- India: the effective reproductive spans, defined as the time between age at marriage and age at sterilization, of successive cohorts of women decreased from 22 years among those who married during the 1960s to 15 years among those who married in the 1970s, to 10 years among those who married in the 1980s and 5 years among those who married in 1990–1996 (Padmadas *et al.*, 2004; Murthy, 2012a).

**Table V:** Temporal trend of women’s reproductive span: UK data.

Year	Menarche	Menopause	Biological span
2006–2010	12.9698	49.6646	36.6948
2012–2013	12.9681	50.2232	37.2551
2014–2018	12.9953	50.3452	37.3499
2019–2021	13.0222	50.3512	37.3290

All data are in years.

**Table VI:** Temporal trend of women’s reproductive span: USA data.

Year	Menarche	Menopause	Biological span
1910–1919	13.12	49.505	36.385
1920–1929	12.93	49.810	36.880
1930–1939	12.76	50.280	37.520

All data are in years.

### Determinants of women’s reproductive span

**Figure 6.**
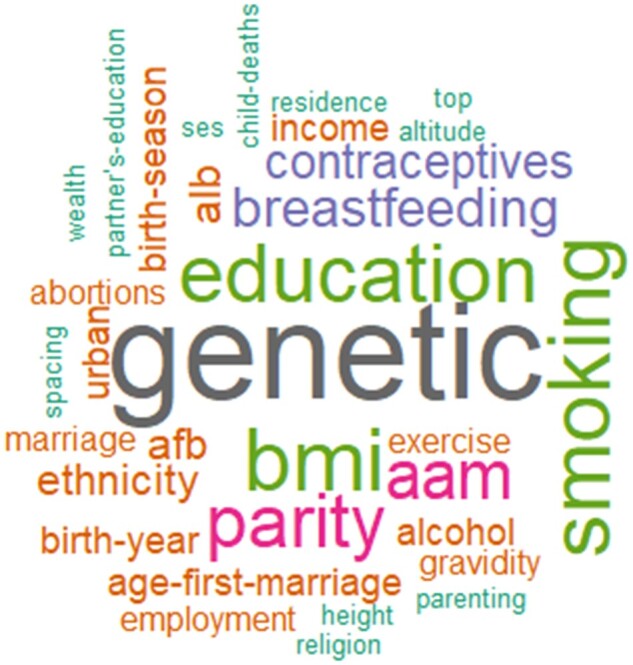
**Word cloud of determinants of women's reproductive span.** All factors shown in the image have been assessed or found to be determinants of women’s reproductive span.

A myriad of factors has been investigated as determinants of women’s reproductive span (Table VII). A word cloud depicts the determinants of women’s reproductive span (Fig. 6).

**Table VII:** Mapping potential determinants of women’s reproductive span.

Concept	Determinant	Association	References
Biological	Genetic:		
	Genes and SNPs	Inconsistent	Hefler *et al.* (2002), Riener *et al.* (2004), Worda *et al.* (2004), Long *et al.* (2006), He *et al.* (2007, 2009b), Kevenaar *et al.* (2007), Mitchell *et al.* (2008), Zerbetto *et al.* (2008), Hartge (2009), He *et al.* (2010), Liu *et al.* (2010), Lu *et al.* (2010), Cerne *et al.* (2011), Chen *et al.* (2012), Carty *et al.* (2013), Tea *et al.* (2013), Pyun *et al.* (2014), Duan *et al.* (2015), Ruth *et al.* (2016), Shi *et al.* (2016) and Fernández-Rhodes *et al.* (2018)
	Telomere length	Increase	Aydos *et al.* (2005)
	Zygosity	Inconclusive	Snieder *et al.* (1998)
	Mothers of Down’s syndrome	No association	Bartmann *et al.* (2005)
	Handedness	No association	Pavia *et al.* (1994)
	Race and ethnicity	Inconsistent	Goodman *et al.* (1985), Wood *et al.* (1985), Menken (1987), Morabia and Costanza (1998) and InterLACE Study Team (2019)
	Non-genetic:		
	Season of birth	Inconsistent	Duarte *et al.* (2017), Si *et al.* (2017) and InterLACE Study Team (2019)
	BMI	Inconsistent	Johnston (2001), Hefler *et al.* (2002), Riener *et al.* (2004), Worda *et al.* (2004), Nichols *et al.* (2006), He *et al.* (2007), Kalichman *et al.* (2007), Dorjgochoo *et al.* (2008), Cerne *et al.* (2011), Forman *et al.*, (2013), Bjelland *et al.* (2018) and Sinha *et al.* (2021)
	Skin fold thickness	No association	Johnston (2001)
	Height	No association	Johnston (2001) and He *et al.* (2007)
	Health status	Inconclusive	Sinha *et al.* (2021)
	Psychosocial stress	May decrease AAM and ANM	Forman *et al.* (2013)
	Arsenic exposure	Decrease	Yunus *et al.* (2014)
	Maternal DDT exposure	Inconclusive	Forman *et al.* (2013)
	Higher polycyclic aromatic hydrocarbons	May decrease	Huang *et al.* (2018)
	Age at menarche	Inconsistent	Snieder *et al.* (1998), He *et al.* (2007), Kalichman *et al.* (2007), Dorjgochoo *et al.* (2008), Chen *et al.* (2010), Liu *et al.* (2010), Mishra *et al.* (2017) and Sharma and Bansal (2018)
	Breastfeeding by subject	Inconsistent	Johnston, 2001; Long *et al.*, 2006; Dorjgochoo *et al.*, 2008; Liu *et al.*, 2010; Cerne *et al.*, 2011; Forman *et al.*, 2013; Sinha *et al.*, 2021
	Breastfed by own mother	May decrease	Johnston (2001)
	Parity	Inconsistent	Johnston (2001), Thomas *et al.* (2001), Long *et al.* (2006), Nichols *et al.* (2006), Kalichman *et al.* (2007), Dorjgochoo *et al.* (2008), Chen *et al.* (2010); Mishra *et al.* (2017) and Sinha *et al.* (2021)
Biological	Marital status	Inconsistent	Johnston (2001), Dorjgochoo *et al.* (2008) and Sinha *et al.* (2021)
	Early age at marriage	Associated	Sharma and Bansal (2018)
	Increase timing between AAM and first livebirth	Increase	Dorjgochoo *et al.* (2008)
	Menstrual irregularities	Decrease	Dorjgochoo *et al.* (2008)
	Average cycle length	No association	Johnston (2001)
	Age at first birth	Inconsistent	Johnston (2001), Thomas *et al.* (2001), Dorjgochoo *et al.* (2008) and Sharma and Bansal (2018)
	Age at last birth	Associated	Dorjgochoo *et al.* (2008) and Sharma and Bansal (2018)
	Age at first and last pregnancy	Associated	Sinha *et al.* (2021)
	Number of pregnancies	Inconsistent	Worda *et al.* (2004), Liu *et al.* (2010) and Cerne *et al.* (2011)
	Weight gain in pregnancy	Inconsistent	Forman *et al.* (2013)
	Birth weight	Inconsistent	Forman *et al.* (2013)
	Birth control	Inconsistent	Johnston (2001), Long *et al.* (2006), Dorjgochoo *et al.* (2008) and Liu *et al.* (2010)
	Abortions	No association	Long *et al.* (2006), Kalichman *et al.* (2007) and Dorjgochoo *et al.* (2008)
	Stillbirths	No association	Dorjgochoo *et al.* (2008)
	Active smoking	Decreases ANM	Johnston (2001), Hefler *et al.* (2002), Riener *et al.* (2004), Worda *et al.* (2004), Long *et al.* (2006), Nichols *et al.* (2006), Dorjgochoo *et al.* (2008), Liu *et al.* (2010), Cerne *et al.* (2011), Fukuda *et al.* (2011), Forman *et al.* (2013), Bjelland *et al.* (2018) and Sinha *et al.* (2021
	Paternal periconceptional smoking	May Decrease ANM	Fukuda *et al.* (2011)
	*In utero* smoking	May decrease AAM and ANM	Forman *et al.* (2013)
	DES exposure	May decrease AAM and ANM	Forman *et al.* (2013)
	Alcohol consumption	No association	Long *et al.* (2006), Dorjgochoo *et al.* (2008), Liu *et al.* (2010) and Cerne *et al.* (2011)
	Physical exercise	Inconsistent	Menken (1987), Long *et al.* (2006) and Dorjgochoo *et al.* (2008)
	Increased total intake of calories, fruits, protein and long-term tea consumption	Increase	Dorjgochoo *et al.* (2008)
	Increased intake of vegetables, soy, fiber, red meat, carbohydrates and fats	No association	Dorjgochoo *et al.* (2008)
	Malnutrition	No association	Menken (1987)
	Low SES	Inconsistent	Menken (1987) and Forman *et al.* (2013)
	Improved living conditions (increased vegetable intake, decreased illiteracy and decreased child labor)	May increase	Thomas *et al.* (2001)
	Higher family income	May Increase	Johnston (2001), Long *et al.* (2006), Dorjgochoo *et al.* (2008) and Sinha *et al.* (2021)
	Current employment	Increase	Johnston (2001)
Biological	Parenting	Inconsistent	Forman *et al.* (2013) and Demakakos *et al.* (2019)
	Higher education	May increase	Johnston (2001) Long *et al.* (2006) Nichols *et al.* (2006), Dorjgochoo *et al.* (2008), Lewington *et al.* (2014), InterLACE Study Team (2019) and Sinha *et al.* (2021)
	Language spoken	No association	Johnston (2001)
	Residence	Inconsistent	Lewington *et al.* (2014) and Duarte *et al.* (2017)
	High altitude	Inconsistent	Beall (1983), Kapoor and Kapoor (1986) and Shaw *et al.* (2018)
Effective	Higher educational level	Decrease	Horne (1989), Padmadas *et al.* (2004), Murthy (2012a,b) and Singh and Singh (2014)
	Increased AAM	Decrease	Wood *et al.* (1985) and Mulder (1989)
	Offspring sex composition	Decrease	Padmadas *et al.* (2004)
	Age cohorts	Inconsistent	Padmadas *et al.* (2004) and Murthy (2012a,b)
	Lack of interspousal communication about family planning	Decrease	Padmadas *et al.* (2004)
	Experiencing pre-marital Hardships	Decrease	Singh and Singh (2014)
	Sterilization	Decrease	Menken (1987)
	Marital dissolution without remarriage	Decrease	Horne (1989)
	Number of child deaths	Increase	Padmadas *et al.* (2004) and Murthy (2012a)
	Fetal loss	Increase	Padmadas *et al.* (2004)
	Termination of pregnancy	Increase	Murthy (2012a,b)
	Increased age at last livebirth	Increase	Horne (1989) and Mulder (1989)
	Contraceptives	Increase	Padmadas *et al.* (2004) and Singh and Singh (2014)
	Marital dissolution with remarriage	Increase	Horne (1989)
	Partner’s education	No association	Murthy (2012a,b)
	Household structure (Nuclear versus non-nuclear families)	No association	Murthy, (2012a)
	Age of first marriage	Inconsistent	Menken (1987), Horne (1989), Murthy (2012a) and Singh and Singh (2014)
	Urban residence	May Decrease	Horne (1989), Padmadas *et al.* (2004) and Murthy (2012a,b)
	Employment	Inconsistent	Murthy (2012a,b)
	Parity	Inconsistent	Horne (1989) and Murthy (2012a)
	Cultural pattern	Associated	Menken (1987)
	Birth interval	Associated	Padmadas *et al.* (2004) and Murthy (2012a)
	Ideal number of offspring	Associated	Murthy (2012a)
	Sex of offspring	Associated	Murthy (2012a)
	Increased wealth index	Associated	Mulder (1989) and Murthy (2012a)
	Religion	Muslims and Christians may have shorter span than Hindus	Padmadas *et al.* (2004) and Murthy (2012a)

AAM, age at menarche; ANM, age at natural menopause; DDT, dichlorodiphenyltrichloroethane; SES, socio-economic status; DES, diethylstilboestrol; SNP, single nucleotide polymorphisms.

#### Biological reproductive span

##### Hereditary factors

Twenty-two studies analyzed genotypic determinants of biological reproductive span. Several genes and intergenic single nucleotide polymorphisms were associated with biological span through age at menarche, age at menopause or both (Table VII).

One study found an association between telomere length and the length of biological span (Aydos *et al.*, 2005).

Other studies investigated the association between biological span and zygosity (Snieder *et al.*, 1998), handedness (Pavia *et al.*, 1994) and mothers of trisomy babies (Bartmann *et al.*, 2005).

##### Ethnicity and racial factors

Japanese women probably have a longer biological reproductive span than Caucasians (InterLACE Study Team, 2019). Gainj women may have a short biological reproductive span (Wood *et al.*, 1985), while Agta Negritos (in the Philippines) may have a longer biological reproductive span than the Dobe Kung (hunter-gatherer women of the Kalahari Desert in Africa) despite a later age at menarche (Goodman *et al.*, 1985). age at menarche was reported to be earlier among the US Black race (Menken, 1987). Asian and African countries have increased age at menarche compared to western countries (Morabia and Costanza, 1998).

##### Environmental factors

Season of birth was not associated with span in one study (Si *et al.*, 2017), while the effect of year of birth varied among studies (Kalichman *et al.*, 2007; Duarte *et al.*, 2017; InterLACE Study Team, 2019).

Changes in body mass index (BMI) were associated with a change in the duration of span in eight studies (Hefler *et al.*, 2002; Riener *et al.*, 2004; Worda *et al.*, 2004; Nichols *et al.*, 2006; Dorjgochoo *et al.*, 2008; Forman *et al.*, 2013; Bjelland *et al.*, 2018; Sinha *et al.*, 2021), while four studies reported no association(Johnston, 2001; He *et al.*, 2007; Kalichman *et al.*, 2007; Cerne *et al.*, 2011). Age at menopause and hence the biological reproductive span was neither associated with skin-fold thickness (Johnston, 2001) nor a woman’s height (Johnston, 2001; He *et al.*, 2007). Psychosocial stress decreases both age at menarche and age at menopause (Forman *et al.*, 2013).

Arsenic exposure was associated with a decrease in biological reproductive span by increasing age at menarche and decreasing age at menopause (Yunus *et al.*, 2014) and higher urinary levels of some types of polycyclic aromatic hydrocarbons is associated with earlier age at menopause (Huang *et al.*, 2018).

The association between the age at menarche and age at menopause was inconsistent (Snieder *et al.*, 1998; He *et al.*, 2007; Kalichman *et al.*, 2007; Dorjgochoo *et al.*, 2008; Chen *et al.*, 2010; Liu *et al.*, 2010; Mishra *et al.*, 2017).

Studies reported inconsistent associations between the duration of biological span and breastfeeding (Johnston, 2001; Long *et al.*, 2006; Dorjgochoo *et al.*, 2008; Liu *et al.*, 2010; Cerne *et al.*, 2011; Forman *et al.*, 2013; Sinha *et al.*, 2021), parity (Johnston, 2001; Thomas *et al.*, 2001; Long *et al.*, 2006; Nichols *et al.*, 2006; Kalichman *et al.*, 2007; Dorjgochoo *et al.*, 2008; Chen *et al.*, 2010; Mishra *et al.*, 2017; Sinha *et al.*, 2021), marital status (Johnston, 2001; Dorjgochoo *et al.*, 2008; Sinha *et al.*, 2021), the age at first birth (Johnston, 2001; Thomas *et al.*, 2001; Dorjgochoo *et al.*, 2008; Sharma and Bansal, 2018), gravidity (Worda *et al.*, 2004; Liu *et al.*, 2010), weight gain in pregnancy (Forman *et al.*, 2013), birthweight (Forman *et al.*, 2013) and the use of contraceptive methods including oral contraceptives and intrauterine device (Johnston, 2001; Long *et al.*, 2006; Dorjgochoo *et al.*, 2008; Liu *et al.*, 2010).

A study suggested that a longer interval between age at menarche and first livebirth may be associated with an increased biological span and that menstrual irregularities maybe associated with changes in biological reproductive span (Dorjgochoo *et al.*, 2008).

The age at last birth (Dorjgochoo *et al.*, 2008; Sharma and Bansal, 2018) and age at first and last pregnancies (Sinha *et al.*, 2021) might be associated with changes in biological reproductive span.

Neither abortions (Long *et al.*, 2006; Kalichman *et al.*, 2007; Dorjgochoo *et al.*, 2008) nor stillbirths (Dorjgochoo *et al.*, 2008) showed an association with biological reproductive span.

Several studies reported an association between smoking and biological reproductive span (Hefler *et al.*, 2002; Worda *et al.*, 2004; Long *et al.*, 2006; Nichols *et al.*, 2006; Dorjgochoo *et al.*, 2008; Liu *et al.*, 2010; Cerne *et al.*, 2011; Fukuda *et al.*, 2011; Forman *et al.*, 2013; Bjelland *et al.*, 2018; Sinha *et al.*, 2021). Most of these studies reported that smoking decreases biological reproductive span (Hefler *et al.*, 2002; Worda *et al.*, 2004; Long *et al.*, 2006; Nichols *et al.*, 2006; Dorjgochoo *et al.*, 2008; Cerne *et al.*, 2011; Fukuda *et al.*, 2011; Forman *et al.*, 2013; Bjelland *et al.*, 2018). Both *in*  *utero* exposure to smoking and paternal periconceptional smoking were associated with earlier age at menopause in offspring who were not actively smoking (Fukuda *et al.*, 2011; Forman *et al.*, 2013).

Three studies reported no association between alcohol and biological reproductive span (Long *et al.*, 2006; Dorjgochoo *et al.*, 2008; Cerne *et al.*, 2011).

Diethylstilboestrol exposure *in*  *utero* decreases both age at menarche and age at menopause, as reported by one study (Forman *et al.*, 2013).

Physical exercise showed a variable association with biological reproductive span. Two studies showed a longer span by increasing age at menopause (Long *et al.*, 2006; Dorjgochoo *et al.*, 2008), while vigorous exercise might shorten the span by delaying age at menarche (Menken, 1987).

Increased total intake of calories, fruits, protein and long-term tea consumption were associated with increased biological reproductive span, while an increased intake of vegetables, soy, fiber, red meat, carbohydrates and fats was probably not associated with changes in biological reproductive span (Dorjgochoo *et al.*, 2008).

Data are inconsistent for the association between low socioeconomic status and biological span (Menken, 1987; Forman *et al.*, 2013). Improved living conditions (increased vegetable intake, decreased illiteracy and decreased child labor) decrease age at menarche, thus increasing biological span (Thomas *et al.*, 2001). Three studies found that higher family income increases biological reproductive span (Johnston, 2001; Long *et al.*, 2006; Dorjgochoo *et al.*, 2008). Current employment was described to have a positive correlation with biological reproductive span (Johnston, 2001).

Two studies report parenting as a determinant of span. One study (Demakakos *et al.*, 2019) found that maternal care, paternal care and maternal over protection are not associated with span, while paternal over protection decreases span. Another study (Forman *et al.*, 2013) reported that paternal absence is associated with early age at menarche.

Higher education might extend the biological reproductive span (Long *et al.*, 2006; Nichols *et al.*, 2006; Dorjgochoo *et al.*, 2008; Lewington *et al.*, 2014; InterLACE Study Team, 2019).

Urban residence might be associated with a longer biological reproductive span (Lewington *et al.*, 2014), while another study found no association (Duarte *et al.*, 2017). Living in high altitude was also investigated in a few studies (Beall, 1983; Kapoor and Kapoor, 1986; Shaw *et al.*, 2018).

#### Effective reproductive span h4

Higher educational level (Horne, 1989; Padmadas *et al.*, 2004; Murthy, 2012a,b; Singh and Singh, 2014), increased age at menarche (Wood *et al.*, 1985; Mulder, 1989), younger women (Padmadas *et al.*, 2004; Murthy, 2012b), experiencing pre-marital hardships (Singh and Singh, 2014), lack of interspousal communication about family planning (Padmadas *et al.*, 2004), offspring sex composition (Padmadas *et al.*, 2004), sterilization (Menken, 1987) and marital dissolution without remarriage (Horne, 1989) were found to decrease effective reproductive span.

Child deaths (Padmadas *et al.*, 2004; Murthy, 2012a), fetal loss (Padmadas *et al.*, 2004), termination of pregnancy (Murthy, 2012a,b), increased age at last livebirth (Horne, 1989; Mulder, 1989), the use of contraceptives (Padmadas *et al.*, 2004; Singh and Singh, 2014) and marital dissolution with remarriage (Horne, 1989) were found to increase effective reproductive span.

The level of a partner’s education (Murthy, 2012a,b) and household structure (nuclear versus non-nuclear families) (Murthy, 2012a) were reported as not associated with effective reproductive span.

The effect of increased age at first marriage was variable among studies. Three studies (Menken, 1987; Horne, 1989; Singh and Singh, 2014) reported that it decreases the effective reproductive span, while one study (Murthy, 2012a) reported the contrary.

Three studies reported that urban residence decreases effective reproductive span (Horne, 1989; Padmadas *et al.*, 2004; Murthy, 2012b), while only one study (Murthy, 2012a) found no association.

Employment (Murthy, 2012a,b) and parity (Horne, 1989; Murthy, 2012a) were also reported to have variable effects on effective reproductive span.

Cultural patterns (Menken, 1987), birth interval (Padmadas *et al.*, 2004; Murthy, 2012a), ideal number and sex of offspring (Murthy, 2012a) and wealth (Mulder, 1989; Murthy, 2012a) are all associated with changes in effective reproductive span.

Concerning ethnicity, Kipsigis (tribe in Kenya) were reported to be associated with a shorter effective reproductive span than Netherlands and US samples, and a comparable effective reproductive span with non-industrialized countries (Mulder, 1989).

Muslims and Christians, compared to Hindus, had a shorter effective reproductive span because of accepting sterilization at a younger age than Hindus (Padmadas *et al.*, 2004; Murthy, 2012a).

In China, the effective reproductive span decreased because of population policies (Lewington *et al.*, 2014).

#### Social reproductive span

We found no studies reporting the determinants of social reproductive span.

### Effects on population demography

Twelve studies reported the effect of reproductive span on demography (Wood *et al.*, 1985; Menken, 1987; Stevenson *et al.*, 1989; Padmadas *et al.*, 2004; Kalichman *et al.*, 2007; Hartge, 2009; Murthy, 2012b; Lewington *et al.*, 2014; Singh and Singh, 2014; Shaw *et al.*, 2018; Gottschalk *et al.*, 2020; Singh *et al.*, 2020). These included six studies of biological reproductive span (Wood *et al.*, 1985; Kalichman *et al.*, 2007; Hartge, 2009; Lewington *et al.*, 2014; Shaw *et al.*, 2018; Gottschalk *et al.*, 2020), five studies of effective reproductive span (Menken, 1987; Padmadas *et al.*, 2004; Murthy, 2012b; Singh and Singh, 2014; Singh *et al.*, 2020) and one study of social reproductive span (Wood *et al.*, 1985).

Two studies reported that the increase in biological span had no effect on the number of births (Kalichman *et al.*, 2007; Gottschalk *et al.*, 2020). In China, an increase in biological span between 1930 and the end of the 20th century occurred, while during a similar period, parity decreased (Lewington *et al.*, 2014).

A systematic review showed that women living at high altitude, compared to those living at low altitude, have a delayed age at menarche and a shorter biological span and this was associated with a lower total fertility (Shaw *et al.*, 2018).

Differences among populations in patterns and dissolution of marriage were associated with changes in total fertility rate. Women with decreased effective reproductive span had a lower fertility rate (Menken, 1987). Four studies reported the impact of effective reproductive span on fertility rate in India (Padmadas *et al.*, 2004; Murthy, 2012b; Singh and Singh, 2014; Singh *et al.*, 2020). The effective reproductive span has decreased in India owing to the rise in legal age of marriage in 1978 and acceptance of earlier sterilization as a method of permanent contraception (Padmadas *et al.*, 2004; Singh *et al.*, 2020). During the same period, fertility rate dropped (Singh and Singh, 2014).

### Effects on fertility services

The available literature lacks primary data examining the impact of reproductive span on the need or utilization of fertility services, including medically assisted reproduction. One narrative review suggested, based on data from the Human Fertilization and Embryology Authority of the UK, that the trend of women being interested in postponing pregnancy to a later age is consistent with the average age of women undergoing IVF or donor insemination in the UK (Barlow, 2011). The narrative review enumerated different approaches that might help to extend the reproductive span, including ovarian tissue cryopreservation and transplantation, oocyte cryopreservation, oocyte donation, embryo cryopreservation, surgical ovarian transposition and suppression of ovarian activity during cancer treatment, modulation of the primordial follicle–primary follicle transition and the possible use of adult somatic cells in the generation of artificial gametes for reproductive use (Barlow, 2011).

## Discussion

This systematic scoping review is the first and most comprehensive attempt to map the extent of research regarding women’s reproductive span. On its own, the review will serve to inform readers on the extent and nature of existing literature in this area, as well as the working definitions, determinants, trends, impact on demographics and assisted reproduction. We identified 67 relevant reports, spanning 120 years, and involving women from 74 countries. We grouped the reproductive span into three concepts, namely biological, social and effective. We summarized key milestones in a woman’s reproductive span which mark the changing life stages. Knowing the typical ages at such events contributes to understanding the changes in family and population. It also helps inform the needs for assisted and other reproductive health services. The review revealed wide variation among reports in the definitions of the start and end of both the biological and the effective reproductive span concepts.

While the extent of the literature on the duration of biological span is sizable and shows minimal trend over decades, the scope of research on the effective reproductive span remains modest despite the considerable trend toward a shorter span.

Several factors have been investigated as determinants of reproductive span with substantial variations in the reported association with women’s reproductive span. This landscape of literature should be read with caution since most of the included literature is cross-sectional, therefore the direction of the association is unknown. Based on this map, rigorous research is warranted to find answers to several questions, for example:

- What are the hypotheses that could be based on these associations?
- What could be the underlying mechanisms of significant associations, if any?

There is insufficient literature on the effect of the current trends in reproductive span on population demographics or assisted reproductive services.

This review has several strengths. These include the extensive search including searching for gray literature. A major challenge that we anticipated as part of this scoping review was that a proportion of the evidence may not be in the bibliographic databases of peer-reviewed journals. For this reason, we also searched the gray and non-bibliographic sources. However, it remains a probability that we may not have captured all relevant sources. Further strengths include adherence to rigorous methods of scoping reviews and the broad inclusion criteria of eligible reports, without restriction by study type, publication status, date or language.

The review has some limitations. A formal assessment of methodological quality of the included studies was not performed because the aim of this review was to provide an overview of the existing evidence base regardless of quality (Peters *et al.*, 2015). Also, the review process did not include a thematic analysis. While we understand the importance of producing a quantitative summary of the association between various determinants and reproductive span, this was neither our aim nor in our planned scoping review methods. Although a comprehensive search was made for existing literature regardless of date, language and peer review status, it is possible that some data were not captured.

This scoping review produced a comprehensive map of the existing literature on women’s reproductive spans. The findings open a window of opportunity to construct clear definitions, generate hypotheses and conduct suitable study designs regarding the determinants of women’s reproductive span, to understand the underlying mechanisms of associations. The wide array of determinants summarized in this scoping review can provide a building block for further research to better understand which of these play a role in the temporal trends of either the biological or the effective reproductive span.

## Supplementary data

Supplementary data are available at *Human Reproduction Open* online.

## Data availability

All data generated or analyzed during this study are included in the published scoping review article and is available upon request from the corresponding author.

## Authors’ roles

A.F.N., G.M. and J.K. conceived the idea for this review. A.F.N. designed the scoping review methods. AF.N., F.E., R.M., M.K., M.E., Y.G.A., Mo.G., M.H.A., M.G., P.E., A.M., M.N., N.A., F.E., A.A.H., N.E., E.S., Y.T., Y.D., N.F., A.A., Y.S. and M.D. collaborated in searching, screening and selecting studies. F.E., R.M., M.K., M.E., Y.G.A., Mo.G., M.H.A., M.G., P.E., A.M., M.N., N.A., F.E., A.A.H., N.E., E.S., Y.T., Y.D., N.F., A.A., M.F.G., M.M., Y.S. and M.D. collaborated in data extraction and synthesis. A.F.N., Y.G.A., F.E., R.M. and M.K. collaborated in writing the first draft of the manuscript. All authors critically reviewed the manuscript resulting in a revision of several drafts. All authors read and approved the final version of the manuscript. G.M and J.K are staff members of the World Health Organization. Views expressed in this manuscript are their own; they do not necessarily represent the views, decisions or policies of the World Health Organization.

## Funding

This work received funding from the UNDP-UNFPA-UNICEF-WHO-World Bank Special Programme of Research, Development and Research Training in Human Reproduction (HRP), a cosponsored programme executed by the World Health Organization (WHO). Grant number 2020/1073913-0.

## Conflict of interest

The authors have no competing interests.

## Supplementary Material

hoac005_Supplementary_Data
