# Supplementary material for: Women’s reproductive span: a systematic scoping review
Source: Hum Reprod Open. 2022 Feb 11;2022(2):hoac005. doi: 10.1093/hropen/hoac005 (PMC8907405; doi:10.1093/hropen/hoac005)
Supplement: hoac005_Supplementary_Data [file hoac005_supplementary_data.docx]

**Supplementary Data**

Search strategies

Pubmed

"menopause/statistics and numerical data"[Mesh] OR "menarche/statistics and numerical data"[Mesh] OR "age at menarche"[tw] OR "age at menopause"[tw] OR "age at natural menopause"[tw] OR "reproductive span"[tw]

Medline

exp menopause/statistics and numerical data/ OR exp menarche/statistics and numerical data/ OR age at menarche.mp. OR age at menopause.mp. OR age at natural menopause.mp. OR reproductive span.mp.

Embase

'menopause/statistics and numerical data'/exp OR 'menarche/statistics and numerical data'/exp OR "age at menarche":ti,ab,de,tn OR "age at menopause":ti,ab,de,tn OR "age at natural menopause":ti,ab,de,tn OR "reproductive span":ti,ab,de,tn

CINAHL

(MH "menopause/statistics and numerical data+") OR (MH "menarche/statistics and numerical data+") OR "age at menarche" OR "age at menopause" OR "age at natural menopause" OR "reproductive span"

Scopus

INDEXTERMS("menopause/statistics and numerical data") OR INDEXTERMS("menarche/statistics and numerical data") OR TITLE-ABS-KEY("age at menarche") OR TITLE-ABS-KEY("age at menopause") OR TITLE-ABS-KEY("age at natural menopause") OR TITLE-ABS-KEY("reproductive span")
